# Study of the Correlations among Some Parameters of the Oxidative Status, Gelatinases, and Their Inhibitors in a Group of Subjects with Metabolic Syndrome

**DOI:** 10.1155/2014/510619

**Published:** 2014-07-10

**Authors:** E. Hopps, R. Lo Presti, M. Montana, B. Canino, M. R. Averna, G. Caimi

**Affiliations:** Dipartimento Biomedico di Medicina Interna e Specialistica, Università degli Studi di Palermo, Via del Vespro 129, 90100 Palermo, Italy

## Abstract

Our aim was to examine some parameters of oxidative status, gelatinases, and their inhibitors and to evaluate their interrelationships in subjects with metabolic syndrome (MS). We enrolled 65 MS subjects, subdivided according to the presence or not of diabetes mellitus. We examined lipid peroxidation (expressed as thiobarbituric acid reacting substances, TBARS), protein oxidation (expressed as carbonyl groups), nitric oxide metabolites (NO_*x*_), total antioxidant status (TAS), MMP-2, MMP-9, TIMP-1, and TIMP-2. We found that MS subjects, diabetics and nondiabetics, showed an increase in TBARS, PC, and NO_*x*_. A significant decrease in TAS was observed only in nondiabetic MS subjects in comparison with diabetic MS subjects. We observed increased concentrations of MMP-2, MMP-9, TIMP-1, and TIMP-2, higher in diabetic subjects. Our data showed a positive correlation between TAS and MMP-2, TAS and MMP-9, and TAS and MMP-9/TIMP-1 and a negative correlation between TBARS and MMP-2 in diabetic MS subjects in the entire group. In MS subjects a prooxidant status and increased levels of gelatinases and their inhibitors are evident although the correlations between oxidative stress and MMPs or TIMPs are controversial and need further investigation.

## 1. Introduction

Today the metabolic syndrome (MS) is considered a public health problem [1]. As it is known, obesity and MS are associated with a low-grade of chronic systemic inflammation, reflected by an increase in circulating leukocytes and by elevated levels of proinflammatory cytokines [2, 3] that contributes to the development of insulin-resistance and atherosclerosis [4] via the activation of nuclear factor-kB pathway [5]. The alterations of the vascular wall start from the endothelial dysfunction and oxidative stress and also an altered matrix metalloproteinases (MMPs) expressions contribute to the consequent remodelling of the basal membrane.

In the last years, we have been interested in the evaluation of the redox balance [6–10] and of the MMPs profile [11, 12] in MS. MMPs are related to atherosclerotic disease and to cardiovascular morbidity and mortality [13–18]; these are endopeptidases responsible for the degradation of several extracellular matrix proteins, such as collagen, laminin, gelatin, and fibronectin [19] which are produced into the vascular wall and are denominated in relation to their target (collagenase, gelatinase, stromelysin, or matrilysin). Gelatinases A and B (MMP-2 and -9) are involved in the vascular remodelling that precedes the atherosclerosis development and also in its worse outcomes [20, 21]. MMP-9 has been discovered in older atherosclerotic lesions [22] and is responsible for fibrosis, matrix degradation, and angiogenesis resulting in plaque instability and rupture [22, 23], while MMP-2 has been correlated with a more stable plaque and with rare haemorrhages [24]. The regulation of MMPs production and activity is complex. Some MMPs (MMP-2) are constitutively expressed on cell surface, while others (MMP-9) are stored in secretory granules and are inducible by exogenous stimuli, such as cytokines, growth factors, and cell-matrix contacts [19, 20]. MMPs are synthesized as precursors (pro-MMP) and they must be activated, to expose the catalytic domain with the Zn^2+^-binding site, by several proteases, such as plasmin, thrombin, chimase, and membrane-type MMP (MT-MMPs) [19, 25], or by S-glutathiolation, S-nitrosylation, and phosphorylation reactions [23].

MMPs and oxidative stress seem to be strongly correlated in subjects with high cardiovascular risk [26–30] and this link has been demonstrated in several experimental models [31–34]. Peroxynitrite (ONOO^−^) activates some MMPs via the S-glutathiolation [31, 34] but, at higher concentrations, can lead to the inactivation of MMP-2 [34]. Also hydrogen peroxide (H_2_O_2_) activates MMP-2 and promotes the expression of MMP-2 and MMP-9 in human venous endothelial cells [35]. Reactive oxygen species (ROS) can influence MMP transcription by means of the activation of the mitogen-activated protein kinase (MAPK), the inhibition of MAPK phosphatase, the inactivation of the histone deacetylase, and the recruitment of different chromatin remodelling factors [36]. MMPs activity is downregulated especially by the four tissue inhibitors of MMP (TIMPs): TIMP-1 inhibits MMP-1, MMP-3, MMP-7, and MMP-9, TIMP-2 inhibits especially MMP-2, and TIMP-3 can inhibit both the gelatinases, while TIMP-4 inhibits MT-1 MMP and MMP-2 activity [14].

In literature, there is no definite information regarding the effects of the oxidative stress on MMPs expression and activity in MS, even if classical cardiovascular risk factors, such as dyslipidemia and diabetes mellitus, increase the gelatinase levels via oxidative stress. In fact, the exposure of endothelial cells to oxidized LDL increases the levels of MT-1 MMP mRNA [37] and the treatment of monocytes-derived macrophages with oxidized HDL induces ROS production, release of TNF-*α*, and an overexpression of MMP-2 and MMP-9 [38]. Also the effects of hyperglycemia on MMP-9 and MMP-2 activity in cultured cells are mediated by ROS [39–41].

Previously, we have focused on the oxidative status [6, 7, 9] and the profile of gelatinases and tissue inhibitors [11, 12] in MS subjects. In this study, our aim was to evaluate, in a group of MS subjects, some parameters of the oxidative status, MMP-2, MMP-9, and their tissue inhibitors in order to investigate their statistical correlations.

## 2. Materials and Methods

We examined 65 subjects (41 men and 24 women; median age 51 yrs; interquartile range 12) selected from those referred to our observation from 2008 to 2011. MS was defined following the International Diabetes Federation (IDF) criteria [1]. The subjects were subsequently subdivided into diabetics (DMS) (22 men and 11 women; median age 59 yrs; IQR 7) and nondiabetics (NDMS) (19 men and 13 women; median age 46 yrs; IQR 9). The baseline characteristics of the two subgroups of DMS and NDMS subjects are described in Table 1. No subjects of both subgroups were taking antioxidants or practising exercise regularly. In the DMS subgroup, only 4 subjects were current smokers while 29 subjects were nonsmokers; in the NDMS subgroup, 11 subjects were current smokers while 21 were nonsmokers. Neither DMS nor NDMS subjects were heavy drinkers. In the subgroup of DMS subjects, diabetes had duration less than 5 years and was treated with diet and oral antidiabetic agents. In all participants, cholesterol and triglycerides were measured by standard enzymatic procedures, HDL-cholesterol after phosphotungstic acid/magnesium chloride precipitation and enzymatic determination of cholesterol, and LDL-cholesterol by the Friedewald formula.

In this group of MS subjects we examined on fasting venous blood the following.

- *Lipid Peroxidation*. The oxidation of polyunsaturated fatty acids was evaluated in plasma by detection of the TBARS, generated by peroxidative processes, which include lipid peroxides and MDA. The evaluation of TBARS was made by fluorimetry, using 1,1,3,3-tetramethoxypropane as standard [42].
- *Protein Oxidation*. The protein carbonyl (PC) content was measured by an enzyme-linked immunosorbent assay (ELISA) kit (BioCell PC test kit, Enzo Life Sciences AG, Switzerland), which uses the classic PC reagent 2,4-dinitrophenylhydrazine (DNP). In brief, plasma samples were incubated with DNP, and then plasma proteins were nonspecifically adsorbed on an ELISA plate. Unconjugated DNP and nonprotein constituents were washed away. The adsorbed proteins were probed with biotinylated anti-DNP antibody, followed by streptavidin-linked horseradish peroxidase. A chromatin reagent was added, and the reaction was stopped by adding an acid solution. Absorbance for each well was measured at 450 nm and related to a standard curve prepared for serum albumin containing increasing proportions of hypochlorous acid-oxidized protein, calibrated colourimetrically. Total protein concentration in plasma samples was evaluated by the method of Lowry et al. [43].
- *Nitric Oxide Metabolites (NO*_*x*_ *). *The NO production was evaluated by a micromethod, which measures the concentration of the NO metabolites, nitrite and nitrate (NO_*x*_).* In vivo* NO has a very short half-life (less than 0.1 sec) and it is converted, through different biochemical pathways, into nitrite, which has a half-life of a few minutes, and the more stable nitrate. Plasma concentrations of nitrate are 90–99% of the total NO metabolites concentration, indicated as NO_*x*_. In the laboratory method adopted by us, nitrate was first converted into nitrite by a nitrate reductase; then nitrite was assessed by spectrophotometry after addition of the Griess reagent [44].
- *Total Antioxidant Status (TAS)*. TAS was obtained using an Assay kit (Calbiochem, La Jolla, CA, USA) which relies on the ability of plasma antioxidant substances to inhibit the oxidation of 2,2-azino-bis(3-ethylbenzthiazoline sulfonic acid) (ABTS) to the radical cation ABTS^•+^ by a peroxidase [45]. The radical concentration was measured by spectrophotometry.
- *Gelatinases and Their Inhibitors*. Plasma concentrations of gelatinases (MMP-2 and MMP-9) and their inhibitors (TIMP-1 and TIMP-2) were determined using, respectively, the Human MMP-2 ELISA and Human MMP-9 ELISA kit (Boster Biological Technology, Ltd.) and the Human TIMP-1 ELISA and Human TIMP-2 ELISA kit (Boster Biological Technology, Ltd.).

The same parameters have been examined in a group of 17 normal subjects (10 men and 7 women; median age 38 yrs; IQR 4), selected from the hospital staff. In this group of control subjects the basal glucose level was 89 (7) mg/dL, total cholesterol level was 200 (40) mg/dL, LDL-cholesterol was 142 (24) mg/dL, HDL-cholesterol was 46 (9) mg/dL, and triglycerides were 65 (36) mg/dL. The mean values of blood pressure in these subjects were 125 (10)/75 (5) mm/Hg; BMI was 26 (4); waist circumference was 98 (13) cm.

The Ethical Committee approved the study and each subject gave informed consent.

## 3. Statistical Analysis

The results were expressed as medians and interquartile ranges (IQR); the differences between MS subjects and normal controls as well as the differences between normal controls and subjects with MS subdivided in agreement with the presence or not of diabetes mellitus were estimated according to the Mann-Whitney test. The study of correlations was performed employing the Spearman rank correlation coefficient.

## 4. Results

Examining the baseline characteristics of subjects with MS we observed a significant decrease in waist circumference (*P* < 0.001), systolic blood pressure (*P* < 0.05), and basal glucose level (*P* < 0.001) and a significant increase in total cholesterol (*P* < 0.001), LDL-cholesterol (*P* < 0.001), triglycerides (*P* < 0.05), and triglycerides/HDL-cholesterol (*P* < 0.05) in NDMS subjects in comparison with DMS subjects (Table 1).

MS subjects showed an increase in lipid peroxidation, protein oxidation, and nitric oxide metabolites (NO_*x*_) (Table 2). In the same group of MS subjects, we found an increase in MMP-2, MMP-9, TIMP-1, and TIMP-2 in comparison with normal subjects; we also observed an increase in MMP-2/TIMP-2 ratio, with no difference regarding MMP-9/TIMP-1 ratio (Table 2). Subdividing the MS group in the two subgroups, we found that the increase in lipid peroxidation, protein oxidation, and NO_*x*_ was similar in DMS and NDMS subjects, while the decrease in TAS was significantly evident only in NDMS subjects, in comparison with normal and DMS subjects (Table 2). We observed also that the plasma concentrations of MMP-2, MMP-9, TIMP-1, and TIMP-2 were significantly increased in the two subgroups in comparison with normal subjects, but in MS subjects with DM the values were higher than in nondiabetics (Table 2). The MMP-2/TIMP-2 ratio was significantly increased in the two subgroups in comparison with normal subjects although its value was lower in NDMS than in DMS subjects (Table 2). The MMP-9/TIMP-1 ratio instead was significantly decreased in MS subjects without DM, not only in comparison with normal subjects but also in comparison with DMS subjects (Table 2).

In normal controls, as well as in the two subgroups of subjects with MS, no statistical correlation was observed among age, parameters of oxidative status, gelatinases, and tissue inhibitors. Examining the linear regression among TBARS, gelatinases, and their inhibitors, we found a negative correlation between TBARS and MMP-2 in DMS subjects (Table 4). No correlation among carbonyl groups, gelatinases, and their inhibitors was evident (Tables 3, 4, and 5) and no relationship among NO_*x*_, gelatinases, and their inhibitors was observed (Tables 3, 4, and 5). A positive correlation between TAS and MMP-2, TAS and MMP-9, and TAS and MMP-9/TIMP-1 ratio in the entire group of MS subjects was found (Table 3).

## 5. Discussion

The results of this research confirm all the data previously published by us [7, 9, 10, 12]: the parameters of the oxidative stress distinguish DMS from NDMS subjects and are altered even in middle-aged MS subjects [10]. Also the gelatinases and their inhibitors discriminate DMS from NDMS subjects and in fact their means were significantly higher in MS diabetic subjects.

Our goal was in particular the evaluation of the statistical correlations between the parameters of the oxidative status and the gelatinases and their tissue inhibitors in MS.

It has been observed that ox-LDLs upregulate MMP-9 expression and reduce TIMP-1 expression in monocyte-derived macrophages [46] and that MDA, which is included in TBARS, is correlated with the MMP-9 activity in subjects with acute coronary syndrome [27]. In this study however the TBARS that reflects lipid peroxidation was negatively correlated with MMP-2 only in DMS subjects.

No correlation was observed among protein oxidation, gelatinases, and their inhibitors, although in experimental models [35] a significant correlation between carbonyl groups and MMP-9 has been described.

We noted especially a positive correlation between TAS and MMP-2, between TAS and MMP-9, and between TAS and MMP-9/TIMP-1 ratio in the entire group of MS subjects. As it is known, TAS includes enzymatic antioxidants (superoxide dismutase, catalase, and glutathione peroxidase) and nonenzymatic antioxidants (uric acid, ascorbic acid, bilirubin, vitamin E, and carotenoids). The examination of the literature data regarding the correlations between antioxidants and MMPs profile shows controversial aspects. In fact, MMP-9 plasma levels are negatively associated with provitamin A carotenoids in a general population [47] while, in experimental models, the deficiency of vitamin A seems to be responsible for a gelatinase decrease without any variations of TIMPs [48]. In subjects with acute stroke the infusion of uric acid induced a decrease of total and active MMP-9 levels [49] and the treatment with antioxidants (polyethylene glycol-superoxide dismutase and N-acetyl-L-cysteine) reduces the MMP-9 activity in plasma and in aortic tissue homogenates of experimental models of diabetes mellitus [39] and the use of tempol (a ROS scavenger) reduces MMP-2 levels and its activity in aortic rings of animal models of renovascular hypertension [50]. Even in experimental models of oxidative stress (obtained with the depletion of glutathione), taurine inhibits MMP-2 activation in cardiac tissues [51]. Differently, the treatment with retinoic acid increases significantly MMP-9 but not MMP-2 [48] and lutein, a carotenoid, enhances MMP-9 synthesis in animal models [52]. Therefore, all these studies do not clarify how in subjects with MS the TAS could be positively related to the gelatinases and their tissue inhibitors and then all these data need further investigation.

With regard to the behaviour of NO_*x*_, its increase in MS is related especially to a nitric oxide overproduction by macrophages, in which the NO synthase activation is caused by cytokines, such as TNF-*α* and IL-1*β* [53–55] that are able to induce also MMPs expression [56–58]. In this study, no statistical correlation was observed among NO_*x*_, gelatinases and their inhibitors in the whole group and in the two subgroups of MS subjects. Keeping in mind that NO production and gelatinases expression are induced by the same cytokines that are increased in MS [59], the inflammatory state could be the link between oxidative stress and MMPs. In addition, it must be considered that, during an inflammatory response, leukocyte infiltration through basal membranes is only possible if these cells produce enzymes that can degrade the extracellular matrix so MMPs, as well as ROS, are crucial effector molecules of inflammatory cells, which play a sure role in atherosclerosis and other chronic inflammatory and metabolic diseases [60].

## 6. Conclusions

There are several data regarding the influence of the oxidative status on the gelatinases and their tissue inhibitors. In this preliminary study concerning a small group of MS subjects, we observed a significant alteration of all these parameters, although from the statistical analysis of the data it is difficult to clarify how the oxidative stress could influence the plasma levels of the gelatinases and their inhibitors. Further investigation seems to be necessary, considering the impact of MS on cardiovascular morbidity and mortality and especially the opportunity of specific therapeutic strategies.

## Figures and Tables

**Table 1:** Medians (interquartile ranges) of the anthropometric profile, blood pressure values, and metabolic pattern in the whole group of MS subjects and in the two subgroups of MS subjects, respectively, with and without diabetes mellitus.

	All MS patients	Diabetic MS patients	Nondiabetic MS patients
Waist circumference (cm)	105 (14.5)	113.0 (16)	100.5 (8)^§^
BMI (Kg/m^2^)	31.95 (4.71)	33.2 (4.4)	31.4 (4.2)
SBP (mmHg)	130 (20)	140 (27.5)	130 (15)^#^
DBP (mmHg)	80 (7.5)	80 (10)	80 (5)
Glycaemia (mg/dL)	101.5 (40.5)	130.5 (80)	91 (13)^§^
Total cholesterol (mg/dL)	207 (74)	186 (50)	227.5 (60)^§^
HDL-cholesterol (mg/dL)	40 (15)	41 (17)	38 (11.5)
LDL-cholesterol (mg/dL)	127.6 (53.6)	109 (47.8)	145 (55.5)^§^
Triglyceridemia (mg/dL)	180 (84)	159 (65)	191.5 (115)^#^
Triglyceridemia/HDL cholesterol	4.31 (3.31)	3.74 (2.41)	5.11 (2.66)^#^

^#^ *P* < 0.05 and  ^§^ *P* < 0.001 versus diabetic MS patients (Mann-Whitney test).

**Table 2:** Medians (interquartile ranges) of gelatinase and inhibitor plasma concentrations in normal controls, in the whole group of MS patients, and in the two subgroups, respectively, with and without diabetes mellitus.

	Control subjects	All MS patients	Diabetic MS patients	Nondiabetic MS patients
TBARS (nmol/mL)	5.71 (2.28)	8.83 (0.70)^‡^	8.99 (0.71)^‡^	8.73 (0.665)^‡^
PC (nmol/mg prot.)	0.470 (0.125)	0.880 (0.240)^‡^	0.880 (0.240)^‡^	0.880 (0.220)^‡^
TAS (mmol/L)	0.910 (0.330)	0.880 (0.300)	0.910 (0.150)	0.720 (0.395)^†§^
NO_*x*_ (*μ*mol/L)	28.05 (24.65)	74.80 (26.52)^‡^	77.10 (24.20)^‡^	67.70 (43.92)^‡^
MMP-2 (ng/mL)	29.19 (6.07)	44.50 (10.46)^‡^	47.84 (10.76)^‡^	39.69 (9.75)^‡§^
MMP-9 (ng/mL)	53.17 (6.57)	105.6 (36.15)^‡^	127.3 (28.7)^‡^	91.50 (11.61)^‡§^
TIMP-1 (ng/mL)	30.99 (2.63)	73.47 (10.31)^‡^	75.66 (8.65)^‡^	68.45 (9.35)^‡§^
TIMP-2 (ng/mL)	87.27 (6.28)	98.77 (14.38)^‡^	99.79 (12.86)^‡^	95.21 (15.32)^†#^
MMP-2/TIMP-2	0.354 (0.080)	0.449 (0.130)^‡^	0.460 (0.104)^‡^	0.391 (0.141)^†#^
MMP-9/TIMP-1	1.720 (0.150)	1.480 (0.345)	1.658 (0.339)	1.347 (0.298)^†§^

*P < 0.05,  ^†^ *P* < 0.01, and  ^‡^ *P* < 0.001 versus control subjects (Mann-Whitney test).

^#^ *P* < 0.01 and  ^§^ *P* < 0.001 versus diabetic MS patients.

**Table 3:** Correlations between oxidative parameters and gelatinases in all MS subjects.

	Versus TBARS	Versus PC	Versus TAS	Versus NO_*x*_
MMP-2	−0.162	−0.043	0.255^#^	−0.077
MMP-9	0.176	−0.025	0.315^#^	0.075
TIMP-1	0.094	−0.067	−0.052	−0.012
TIMP-2	0.126	−0.199	0.067	0.071
MMP-2/TIMP-2	−0.206	0.035	0.196	−0.100
MMP-9/TIMP-1	0.133	−0.017	0.320^§^	0.097

^#^ *P* < 0.05;  ^§^ *P* < 0.01 (Spearman's rank correlation).

**Table 4:** Correlations between oxidative parameters and gelatinases in diabetic MS subjects.

	Versus TBARS	Versus PC	Versus TAS	Versus NO_*x*_
MMP-2	−0.363^#^	−0.240	−0.235	−0.144
MMP-9	0.180	−0.144	−0.057	0.295
TIMP-1	0.002	0.000	−0.287	0.079
TIMP-2	0.224	−0.266	0.148	0.287
MMP-2/TIMP-2	−0.283	−0.100	−0.263	−0.149
MMP-9/TIMP-1	0.167	−0.136	0.028	0.216

^#^ *P* < 0.05 (Spearman's rank correlation).

**Table 5:** Correlations between oxidative parameters and gelatinases in nondiabetic MS subjects.

	Versus TBARS	Versus PC	Versus TAS	Versus NO_*x*_
MMP-2	−0.346	0.097	0.207	−0.167
MMP-9	−0.041	−0.021	0.236	−0.163
TIMP-1	0.055	−0.174	−0.289	−0.167
TIMP-2	−0.067	−0.154	−0.193	−0.140
MMP-2/TIMP-2	−0.280	0.195	0.316	−0.077
MMP-9/TIMP-1	−0.104	0.084	0.317	−0.065
